# Aircraft and Ship Velocity Determination in Sentinel-2 Multispectral Images

**DOI:** 10.3390/s19132873

**Published:** 2019-06-28

**Authors:** Henning Heiselberg

**Affiliations:** National Space Institute, Technical University of Denmark, 2800 Kongens Lyngby, Denmark; hh@space.dtu.dk; Tel.: +45-4525-9760

**Keywords:** Sentinel-2, multispectral, temporal offsets, ship, aircraft, velocity, altitude, parallax, jet stream

## Abstract

The Sentinel-2 satellites in the Copernicus program provide high resolution multispectral images, which are recorded with temporal offsets up to 2.6 s. Moving aircrafts and ships are therefore observed at different positions due to the multispectral band offsets, from which velocities can be determined. We describe an algorithm for detecting aircrafts and ships, and determining their speed, heading, position, length, etc. Aircraft velocities are also affected by the parallax effect and jet streams, and we show how the altitude and the jet stream speed can be determined from the geometry of the aircraft and/or contrail heading. Ship speeds are more difficult to determine as wakes affect the average ship positions differently in the various multispectral bands, and more advanced corrections methods are shown to improve the velocity determination.

## 1. Introduction

Surveillance for marine and air space situation awareness is essential for monitoring and controlling traffic safety, piracy, smuggling, fishing, irregular migration, trespassing, spying, icebergs, shipwrecks, the environment (oil spill or pollution), etc. Dark ships and aircrafts are non-cooperative vessels with non-functioning transponder systems such as the automatic identification system (AIS) for ships or automatic dependent surveillance (ADS-B) for aircrafts. Their transmission may be jammed, spoofed, sometimes experience erroneous returns, or simply turned off deliberately or by accident. Furthermore, AIS and ADS-B land based and satellite coverage is sparse at sea and at high latitudes. Therefore, other non-cooperative surveillance systems as satellite or airborne systems are required.

The Sentinel-2 satellites under the Copernicus program [1,2,3] carry multispectral imaging (MSI) instruments that provide excellent and freely available imagery with pixel resolutions down to 10 m. The orbital period is 5 days between the Sentinel-2 (S2) satellites A + B, but as the swaths from different satellite orbits overlap at higher latitudes, the typical revisit period for each satellite is two or three days in Europe and almost daily in the Arctic. S2 MSI has the potential to greatly improve the marine and airspace situational awareness, especially for non-cooperative ships and aircrafts.

Ship detection, recognition, and identification in optical satellite imagery has been studied in a number of papers with good results [4,5,6,7,8,9,10]. The resolution and sensitivity are generally better and the number of multispectral bands is larger, but clouds reduce the continuous coverage. Ship positions, and their length, breadth, form and heading can be determined accurately and the multispectral reflections can be fingerprints for ID. Ship speeds have only been determined from satellite imagery in a few cases where Kelvin wakes are observed [7].

Detection, tracking and speed determination of vehicles on ground has been studied in video sequences and images recorded with time intervals fx. by change detection. Heights of tall buildings or altitudes of clouds [11,12,13] and other static or slow moving objects have been determined by shadow lengths or parallax methods, when the images are recorded from a flying platform as an aircraft, drone or satellite with time delay imaging or from two platforms.

Recently, parallax effects were observed for aircrafts and their condensation trails (contrails) in Sentinel-2 color images, referred to as “plainbows” [14]. The work presented here is novel when it comes to exploiting the temporal offsets in Sentinel-2 MSI, and specifically for determining aircraft velocities and altitudes, and ship speeds. We consider this work as the first analysis of such effects due to temporal offsets, as we have not been able to find any studies with scientific analyses or applications. In this work, we outline the basic physics for moving objects in satellite multispectral images with temporal offsets, the parallax effects and influence of jet streams. We primarily consider aircrafts and ships, but the analysis also applies in principle to all kinds of moving vehicles including cars and helicopters, and also clouds, auroras, etc. The basic formulas are derived, and as proof of principle, we show a number of representative examples for both aircrafts and ships. From Sentinel-2 multispectral images with known temporal offsets we calculate the resulting speed, heading, altitudes, etc. Subsequently, we test our results by comparing to data from the navigation systems ADS-B or AIS.

In Section 2 we analyze how the temporal offset affects moving objects in S2 multispectral images and include the parallax effect for aircrafts as well as the effect of jet streams. After a description of the S2 MSI offset times and resulting apparent velocities, we calculate aircraft velocities and altitudes from S2 multispectral images in Section 3. In Section 4 we turn to ship velocities, which are not affected by parallax but are slower and more difficult to determine accurately due to long wakes. We describe a simple but effective correction method, which improves the speed calculations considerably.

In the summary and outlook we suggest ways to improve the model by better position determination and understanding of spectral dependence of object reflections [15]. In addition, calculations for a large number of ships and aircrafts are required for better statistics and improving the model by fine-tuning the parameters. This could also lead to an annotated database useful for machine learning methods.

## 2. Satellite Images and Method of Analysis

**Figure 1:**
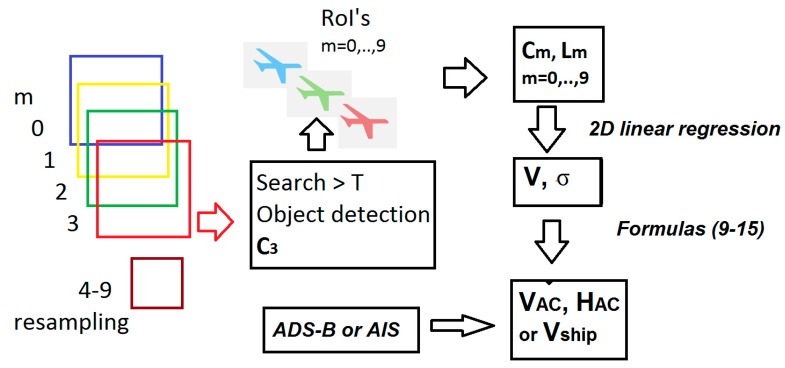
Flow chart of algorithm: For a given scene the m = 0, …, 9 high resolution S2 multispectral images are selected. Search and detection of objects above a threshold T is done for the red band m = 3. For each object, a region of interest (RoI) around the object center ${\vec{c}}_{3}$ is chosen for all ten images, where m = 4–9, are resampled to 10 m pixel size. The center ${\vec{c}}_{m}$ and length ${\vec{L}}_{m}$ are calculated for m = 0, ..., 9. From 2D linear regression (3) of the variance σ, the apparent velocity $\vec{V}$ is found and inserted in (9–15), whereby the aircraft velocity and altitude or ship velocity is found. By comparing to ADS-B or AIS, the velocities, altitudes and positions are validated.

The S2 multispectral images were analyzed using dedicated software developed specifically to detect ships and aircrafts in large images with different pixel resolutions and determine their precise position and orientation as described in [7,8]. As detection is not the focus of this paper, we mainly describe the MSI temporal offsets and how they affect the multispectral images for velocity calculations. A flow chart for the algorithm is shown in Figure 1.

### 2.1. Sentinel-2 Multispectral Images

S2 carries the Multispectral Sensor Imager [1,2,3] that records images in 13 multispectral bands (see Table 1) with different resolutions and time offsets. As we are interested in small object detection and tracking, we focused on analyzing the high resolution images, the 4 bands with 10 m and the 6 bands with 20 m pixel resolution. These are mega- to giga-pixel images with 16 bit grey levels.

**Figure 2:**
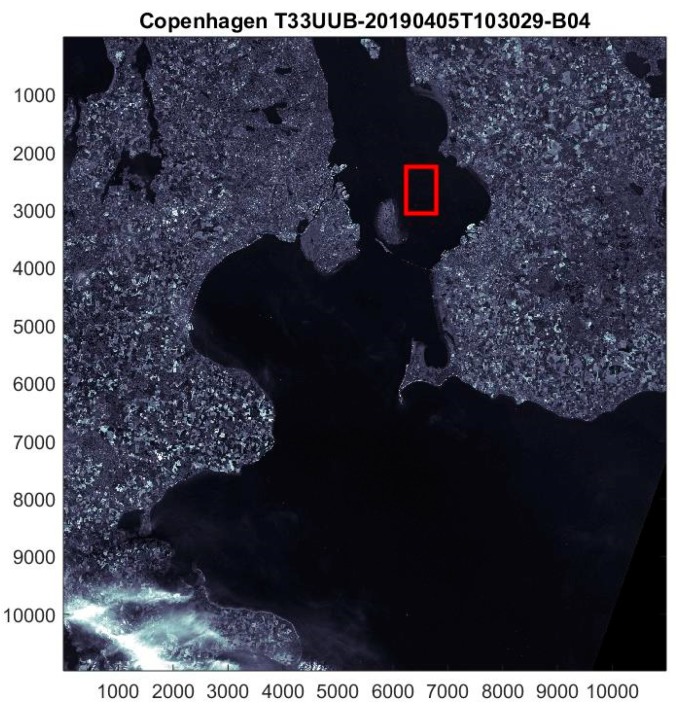
East part of Denmark in the red band B4 (m = 3) from 5 April 2019, 10:30 a.m. UTC. The red box is a ROI in the strait of Øresund surrounding Copenhagen airport.

We analyzed several S2 level 2A processed images from 2019 covering Copenhagen airport in Denmark, see Figure 2, and the Channel between Amsterdam and London. These images are convenient because several aircrafts are usually present after takeoff or before landing. In addition, there are a larger number of ships present in the strait of Øresund surrounding the airport and in the Channel.

In the S2 multispectral images $\;I_{m}\left(i,j\right)$, the spatial coordinates $\vec{r}=\left(x,y\right)$ are the pixel coordinates (*i*,*j*) multiplied by the pixel resolution *l* = 10 m, 20 m or 60 m as given in Table 1 for the 13 bands. The 10 high resolution multispectral images with pixel size 10 m or 20 m are ordered according to temporal offset $t_{m}$, *m* = 0, 1, ..., 9. As shown in Table 1, they range from 0 s to 2.085 s in temporal offset. Due to the odd and even detector array in MSI, the offsets are either delayed or advanced, respectively. The imaging sequence is such that the offsets are reversed in stripes along track within the image [13].

### 2.2. Object Detection and Position Determination

To detect an object, its reflection must deviate from the background. For proving the principle of velocity determination, we chose for simplicity a region of interest with sea background, which is usually darker than the objects and therefore makes detection easier. The multispectral variant background over land will require a more elaborate detection algorithm, but has the potential of determining velocities of driving vehicles as well.

**Figure 3:**
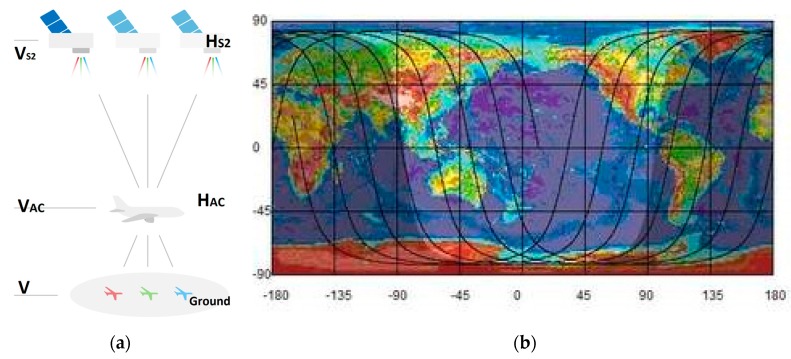
(**a**) Illustration of the satellite-aircraft-ground parallax effect. (**b**) Earth map with projected satellite orbits. The orbits cross the vertical lines (latitudes ϕ) at angles $\theta_{S2}$.

**Figure 4:**
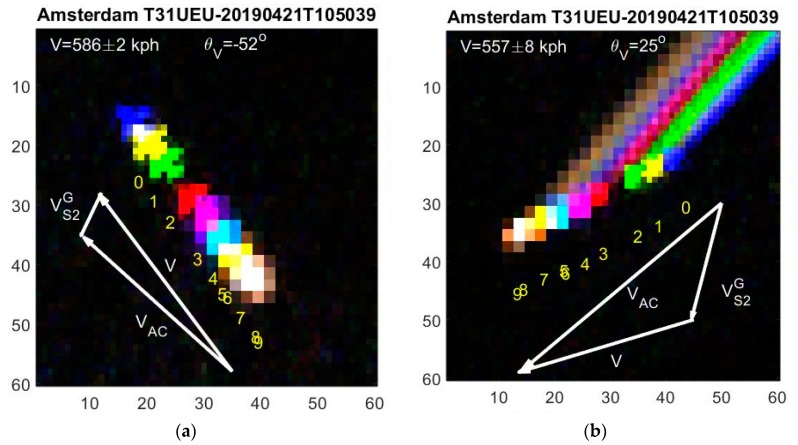
Planebows. Scale in pixels. (**a**) A slow and low flying aircraft near Amsterdam airport after takeoff recorded in 10 multispectral images with offsets. Red, green and blue are true colors whereas the seven remaining colors are overlayed with false colors, e.g., m = 1 is yellow. Numbers 0, ..., 9 indicate the central aircraft position in each band (but moved 10 pixels down). The triangle shows the apparent ($\vec{V}$), aircraft (${\vec{V}}_{AC}$ ) and satellite (${\vec{V}}_{S2}^{G}={\vec{V}}_{S2}H_{AC}/H_{S2}$ ) velocity vectors. (**b**) Planebow of a fast flying aircraft at high altitude near Amsterdam with strong contrails from each wing motor.

**Figure 5:**
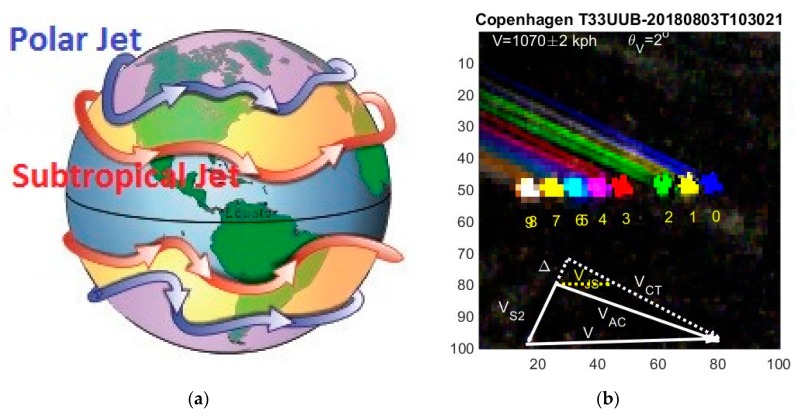
(**a**) Illustration of Earth’s meandering polar and subtropical jet streams. (**b**) An aircraft over Copenhagen where the contrails are affected by the polar jet stream (see text).

When the sea covers more than half of the image after land removal, the median reflection value provides an accurate and robust value for the average background. For detection, we chose the red band *m* = 3 (see Figure 2) because it has high resolution and average time offset such that temporal offset objects will appear around the red center (see Figure 3, Figure 4 and Figure 5). In addition, solar reflections from ships and aircrafts generally have high contrast in red with respect to the sea background. For each object a small region, e.g., 100 × 100 pixels or smaller, is extracted around the central object coordinate, such that it covers the object extent including movement, wakes or contrails. The same 1 km × 1 km region is then extracted for the 10 high resolution bands *m* = 0, 1, 2, 3 with spatial resolution 10 m and *m* = 4, ..., 9 with 20 m. The latter are corrected for the different resolutions. For each band the median value is chosen as the background.

The pan-sharpening technique [7,16] for increasing the resolution in lower resolution images can only be applied to static images. As moving objects change pixel position in the multispectral images due to the temporal offset, we could not apply pan-sharpening in this analysis.

For each multispectral image, the object is defined spatially by the pixels with reflections above the background value plus a threshold T, which depends on target type as will be discussed below. The central object position ${\vec{c}}_{m}=(x,y)$, length $L_{m}$, breadth $B_{m}$ and orientation/heading angle $\theta_{m}$ are calculated in each band *m* = 0, ..., 9 by weighting the object pixels with their reflection $I_{m}\left(i,j\right)$ and calculating the first moments in *i* and *j*, as described in detail in [7].

Unfortunately, ship wakes and aircraft contrails can corrupt the position determination considerably. Both generally move the central position backwards with respect to vessel direction by an amount that varies with the band. At the same time, the object length is extended. We corrected for this effect to the first order by adding the distance from the average center position to the ship front, which is half of the object length $L_{m}$, in the vessel heading direction, i.e., the vector ${\vec{L}}_{m}=L_{m}(\cos\theta_{m},\sin\theta_{m})$.

(1) $${\vec{r}}_{m}={\vec{c}}_{m}+\frac{1}{2}{\vec{L}}_{m}$$

This position is now at the front of the object in each band *m* as shown in the images below.

### 2.3. Multispectral Temporal Offsets and Velocity Determination

We define the apparent velocity as the change in position as observed in the multispectral images divided by the band dependent time delay. An object moving with apparent velocity $\vec{V}$ will ideally be recorded in band *m* at position

(2) $${\vec{r}}_{m}∼{\vec{r}}_{V}+\;\vec{V}\cdot t_{m}$$

here, ${\vec{r}}_{V}$ is the vessel position at zero temporal offset—ideally the blue band *m* = 0 for which $t_{0}=0$. For ships, the apparent velocity $\;\vec{V}$ is simply the vessel speed and direction, whereas for aircrafts, the parallax effect due to satellite motion must be included as will described below. Currents and jet streams also influence V.

For example, the aircrafts shown in Figure 4 and Figure 5 fly with apparent speeds V ~ 200 m/s, and move a distance of ~400 m or 40 pixels in the time interval of 2.085 s. Consequently, the aircrafts show up as ten “pearls on a string” when the high resolution multispectral bands are plotted all together in a (false) color image. 

The object positions ${\vec{r}}_{m}$ that are calculated for each multispectral image *m* = 0, ..., 9 will generally scatter around the linear prediction of Equation (2). We define the variance as the mean square average of distance deviations

(3) $$\sigma^{2}=\frac{1}{10}\overset{9}{\underset{m=0}{\sum }}\;\;{\left({\vec{r}}_{m}-{\vec{r}}_{V}-\vec{V}\cdot t_{m}\right)}^{2}$$

By minimizing this variance, which is equivalent to a two-dimensional linear regression, we obtain the best fit values for vessel position ${\vec{r}}_{V}$ and the apparent velocity vector $\vec{V}=V\left(\cos\theta_{V},\sin\theta_{V}\right)$. An estimate for the uncertainty in apparent velocity is the lowest standard deviation in distance divided by the temporal interval

(4) $$\sigma_{V}=\sigma /t_{9}$$

Typically, the positions are accurate up to a pixel size *l* or less in each multispectral image. The two-dimensional linear regression fit of ${\vec{r}}_{m}$ vs. *t_m_* therefore has a standard deviation less than $\sigma ∼l/\sqrt{10}$. Dividing by the temporal offset interval *t*_9_ = 2.085 s, we obtain the uncertainty for the apparent velocity of $\sigma_{V}=l/t_{9}\sqrt{10}$ ~5–10 kph. This is comparable to speeds of slow ships, whereas typical aircraft cruise velocities are 800–1000 kph, and gives a relatively accurate aircraft velocity determination.

### 2.4. Comparison to AIS and ADS-B

By matching positions of aircrafts from ADS-B and ships from AIS at the same time and positions, we can identify the vessels and compare size, heading, velocities and altitudes. Unfortunately, the positioning systems sometimes lag or the updating is delayed or is infrequent. Therefore, we find that ships and aircraft do not always match precisely at the correct position and time. In addtion, the S2 overflight time included in the file name is not the local image recording time. In Figure 2, the Copenhagen regions are recorded five minutes later than the time 10 h 30 m 29 s given in the file name, and the local recording time is 14 s later from north to south for the descending track.

## 3. Aircraft Velocities

For low flying aircrafts and ships the parallax effect is negligible and the apparent velocity $\;\vec{V}\;$ is just the aircraft velocity ${\vec{V}}_{AC}$. For high altitude aircraft, we need to consider the satellite orbit, velocity and viewing angle in order to correct for the parallax effect. In addition, the jet stream must be considered as it affects the contrails.

.

### 3.1. Satellite Direction w.r.t. Ground

We used standard mathematical and celestial convention where angles are measured from the equator counter-clockwise. In navigation, angles are measured from the North Pole clockwise and thus differ by 90° and angular direction.

The S2 satellites fly in a sun-synchronous orbit at mean altitude H_S2_ = 786 km with speed V_S2_ = 7.44 km/s. Their polar orbit is slightly retrograde, descending with inclination angle i = −98.62° on the dayside. Due to Earth’s curvature, the orientation of the satellite track $\theta_{S2}$ with respect to latitude ϕ is (see Figure 3b)

(5) $$\cos\theta_{S2}=\cos i/\cos\phi$$

At the equator, ϕ = 0° and $\theta_{S2}=i$, but at maximum polar S2 latitude ϕ=180°−i=81.38°, the S2 satellite flies straight west, i.e., $\theta_{S2}=\pm 180{}^{\circ}$. For the images around Copenhagen, ϕ≃55° and we find $\theta_{S2}≃-105{}^{\circ}$. At Amsterdam, ϕ≃52,5° and $\theta_{S2}≃-104{}^{\circ}$. The resulting satellite velocity is ${\vec{V}}_{S2}=V_{S2}\left(\cos\theta_{S2},\sin\theta_{S2}\right)$, w.r.t. ground.

### 3.2. Parallax Effect

The satellite movement during the multispectral temporal delays causes a parallax effect (see Figure 3a) that moves objects at an altitude H northeast-wards in direction $-\theta_{S2}$ with respect to the ground. Stationary objects such as tall buildings, clouds, balloons, stalling aircrafts, etc. will be moved by an apparent velocity $\vec{V}=-{\vec{V}}_{S2}\cdot H/H_{S2}$ with respect to ground due to their parallax. Determining *V* from a linear regression of Equation (3), we find the object altitude

(6) $$H=\;\frac{V}{V_{S2}}\cdot H_{S2}$$

The parallax has recently been exploited for determining altitudes and movement of, for example, volcanic plumes [11,12].

The parallax effect moves the flight paths opposite to the satellite direction, and separates each multispectral band such that an aircraft (with its contrails) appears as a multispectral rainbow, when plotted in a false color image as shown in Figure 4 and Figure 5. Previously [14], the RGB contrails were named “plainbows”. We named our ten multispectral contrails as a “planebows”. Contrails are usually observed at high altitudes 7.5–12 km.

### 3.3. Moving Objects

When the object moves, its velocity must be added to the apparent velocity. In the absence of wind, a moving object, such as an aircraft at altitude $H_{AC}$ with velocity ${\vec{V}}_{AC}=V_{AC}\left(\cos\theta_{AC},\sin\theta_{AC}\right)$, will appear to have velocity

(7) $$\vec{V}={\vec{V}}_{AC}-{\vec{V}}_{S2}\cdot \frac{H_{AC}}{H_{S2}}\;\;$$

or

(8) $$\left(\begin{array}{c} cos\;\theta_{AC}\\ sin\;\theta_{AC} \end{array}\right)V_{AC}=\;\left(\begin{array}{c} cos\;\theta_{V}\\ sin\;\theta_{V} \end{array}\right)V+\left(\begin{array}{c} \cos\theta_{S2}\\ \sin\theta_{S2} \end{array}\right)V_{S2}\cdot \frac{H_{AC}}{H_{S2}}$$

The heading of the aircraft $\theta_{AC}$ is given by the aircraft orientation angles $\theta_{m}$, *m* = 0, ..., 9 as described in Section 2.2 and [7]. The aircraft heading angle $\theta_{AC}$ must be determined independently for the ten multispectral bands $\theta_{m}$. The four high resolution bands generally provide a consistent and robust average aircraft heading angle $\;\theta_{AC}$. When contrails are visible (see Figure 3b), the contrail and aircraft directions are the same $\theta_{CT}=\theta_{AC}$, and they provide a more accurate heading. 

From the two equations in (8), we obtain the aircraft velocity

(9) $$V_{AC}=\;\frac{sin(\theta_{V}-\theta_{S2})}{sin\left(\theta_{AC}-\theta_{S2}\right)}\cdot V$$

and the aircraft altitude

(10) $$H_{AC}=\;\;\frac{sin(\theta_{V}-\;\theta_{AC})}{sin\left(\theta_{AC}-\theta_{S2}\right)}\cdot \frac{V}{V_{S2}}\cdot H_{S2}$$

These relations can also be obtained from simple sine relations for the triangles in Figure 4 and Figure 5.

When the aircraft and satellite directions are parallel, $\theta_{S2}=\theta_{AC}$, the aircraft velocity and altitude cannot be determined separately. At zero altitude (as will be discussed below for ships) there is no parallax effect so that $\theta_{AC}=\theta_{V}$ and $\;V_{AC}=V$.

Note that Equations (9) and (10) are invariant to the orientation of the coordinate system as only relative angles appear. They are also clock- vs. counter clockwise invariant.

In Figure 4a, a slow and low flying aircraft is shown after take-off from Amsterdam. From linear regression of Equation (3), we find its apparent velocity V = 586 ± 2 kph and direction $\theta_{V}=128{}^{\circ}$. The aircraft orientation angle $\theta_{AC}=133{}^{\circ}$ is determined by the object orientation. From Equations (9) and (10), we find an aircraft speed of $V_{AC}=552$ kph, at altitude $H_{AC}=1.764$ m. According to live flight tracking, ADS-B, the aircraft at that time and position, has a speed of 507 kph at altitude 1.875 m. Considering delays in the flight tracking updates and neglecting wind speeds, the agreement is fair.

In Figure 4b another aircraft with strong contrails is captured near Amsterdam, where we expect little jet stream. Thus the long contrails show the aircraft heading $\theta_{CT}=\theta_{AC}=-134{}^{\circ}$. The contrails do, however, corrupt the determination of the aircraft central positions, and we therefore remove them in the object images by setting the threshold T sufficiently high above the contrail but below the aircraft reflections. The central object positions in each band can then be used for determining the aircraft positions ${\vec{r}}_{m}$ as shown in Figure 4b. The resulting apparent velocity is *V* = 557 ± 8 kph, and the apparent direction $\theta_{V}=-155{}^{\circ}$. From Equations (9) and (10), we find an aircraft speed of $V_{AC}=\;$855 kph and altitude $H_{AC}=11.231$ m. According to the flight tracking system, an aircraft at that time and position has speed $V_{AC}=\;$833 kph and altitude $H_{AC}=11.582$ m, both in good agreement with our calculations.

The large apparent velocities *V* can in turn be exploited for a search for aircrafts in S2 images as they are the only fast moving objects.

### 3.4. Jet Stream and Contrails

The polar jet stream circulates eastward in a meandering way as illustrated in Figure 5a. It lies between latitudes 50–60° at altitudes 9–12 km, and are a few hundred km wide. The jet typically has a speed of ~100 kph but can exceed 400 kph. Flight information systems show that aircrafts benefitting from the jet stream typically fly a hundred kph faster east- than westwards. 

The jet stream (and winds in general) with velocity ${\vec{V}}_{JS}$ will sweep an aircraft and its contrails along. The aircraft orientations $\theta_{m}$ are the same as the contrail direction $\theta_{CT}$. The aircraft orientation in the image no longer matches the true aircraft heading $\theta_{AC}$ with respect to ground as shown in Figure 5b. If the contrail angle is used in Equations (9) and (10) for the aircraft angle, it can erroneously lead to supersonic aircrafts flying well above the altitude of most commercial airliners. Such extreme values only apply to the Concorde and a few other aircrafts. Fighter jets can be excluded in Figure 5b as the size of the aircraft is too large.

In order to correct for wind speeds, we introduce two auxillary quantities (see Figure 5b), namely the velocity in the contrail direction

(11) $$V_{CT}=\;\frac{sin(\theta_{V}-\theta_{S2})}{sin\left(\theta_{CT}-\theta_{S2}\right)}\cdot V$$

and the jet stream correction to the parallax effect

(12) $$\Delta =\;\frac{sin(\theta_{JS}-\theta_{CT})}{sin\left(\theta_{CT}-\theta_{S2}\right)}\cdot V_{JS}$$

The aircraft altitude is then (see Equation (10))

(13) $$H_{AC}=\frac{H_{S2}}{V_{S2}}\;(\frac{sin(\theta_{V}-\theta_{CT})}{sin\left(\theta_{CT}-\theta_{S2}\right)}\cdot V-\Delta )$$

The aircraft velocity can be determined from

(14) $$V_{AC}^{2}=V_{CT}^{2}+\Delta^{2}-2V_{CT}\cdot \Delta \cdot cos\left(\theta_{CT}-\Theta_{S2}\right)$$

Finally, the aircraft heading angle can be determined from Equation (9).

In Figure 5b the aircraft engines under each wing create two long contrails with angle $\theta_{CT}=-24{}^{\circ}$. The apparent velocity is V = 1070 ± 2 kph with direction $\theta_{V}=2{}^{\circ}$. From Equation (11) we find $V_{CT}=1035$ kph. Assuming that the jet stream heads east $\theta_{JS}=0{}^{\circ}$ with speed $V_{JS}=200$ kph, we obtain Δ=82 kph, $H_{AC}=11.514$ m, $V_{AC}=1.025$ kph and $\theta_{AC}=-19{}^{\circ}$. The aircraft speed relative to the jet stream is thus 825 kph. These numbers are compatible with normal aircraft cruising altitude and speed, however, the unknown jet stream speed was fitted.

## 4. Ship Velocities

Low objects on the surface of the Earth, such as ships, have no parallax. In addition, ocean currents are usually slow and can be neglected. The apparent velocity is then simply the ship velocity

(15) $$V_{Ship}=\;V$$

**Figure 6:**
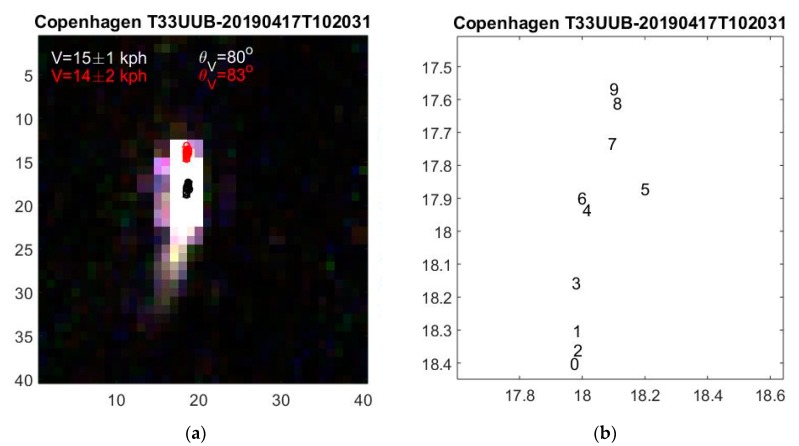
(**a**) Ship with short wake. As all ten multispectral ship images almost overlap, they appear white. The central positions shown with black numbers *m =* 0, ..., 9 are expanded in (**b**). The temporal ordering appears approximately correct.

**Figure 7:**
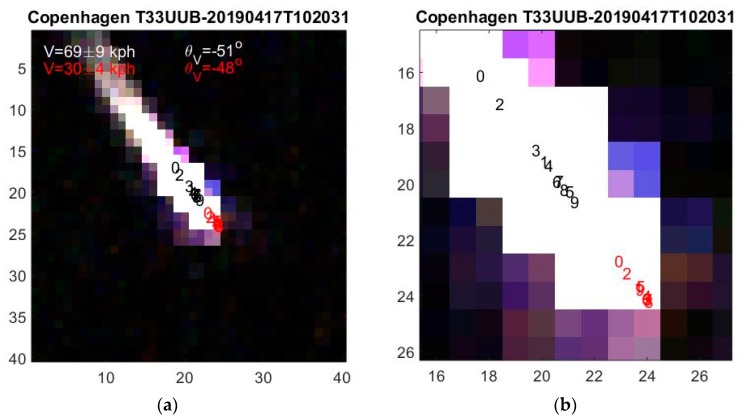
(**a**) As Figure 6 but for a fast ship creating a long wake, which corrupts the temporal ordering. Adding ship lengths L_m_ to central position as shown with red numbers in (**b**) improves the temporal ordering and velocity determination.

Likewise, the vessel heading angle is the apparent direction $\;\theta_{V}$ and is the same for all bands. By overlaying all multispectral images as shown in Figure 6 and Figure 7, the heading angle $\theta_{V}$ can be determined accurately, especially for large ships and/or when ship wakes are long.

Ships sail much slower than aircrafts, typically ~10 m/s (19.4 knots or 36 kph). Therefore, the ships in Figure 5 and Figure 6 move less than two pixels in the temporal offset interval *t*_9_ = 2.085 s, which requires accurate determination of ship positions. 

### 4.1. Short Wakes

When ship wakes are short, they have less effect on the estimated central positions and both the central and the corrected positions of Equation (1) can be used for determining the ship velocity. An example is shown in Figure 6 where ${\vec{c}}_{m}$ and ${\vec{r}}_{m}$ are plotted with black and red numbers, respectively. Both are temporally ordered correctly and yield the ship velocity V=15±1 kph. According to AIS, a ship at that time and position is sailing at a speed of 15 kph in the same direction.

### 4.2. Wake Corrections

Unfortunately, ship wakes can be longer than the ship and we find that they can corrupt the position determination considerably. Ship wakes move the apparent central position backwards with respect to vessel direction by an amount that typically is larger for the lower wavelengths. This is observed in Figure 7 where the ordering is not temporal but rather spectral, i.e., according to band wavelength as: *m* = 0, 2, 3, 4, 6, 7, 1, 8, 5, 9 (see Table 1). Wake reflection seems to decrease gradually towards the infrared [9,10,15]. If one simply uses the central positions for determining V, one obtains a ship speed of 69 ± 9 kph, which is much too large. Using the front positions of Equation (1), their ordering is closer to temporal and the ship speed is only 30 ± 4 kph. According to AIS, a ship at that time and position has a speed of 25 kph in the same direction. The length correction to central positions not only improves the accuracy of the velocity determination but also reduces the uncertainty.

The uncertainty in velocity is typically 5–10 kph, which sets the limit on how slow ship speeds can be determined. To improve the position accuracy, the more advanced calculation of ${\vec{r}}_{m}$ and study of the spectral dependence of wakes is required.

The two examples in Figure 6 and Figure 7 may give the wrong impression, that faster ships create longer wakes, which is not always the case. Wake length may depend on e.g., ship type and size, surface winds and background. The temporal offsets are therefore useful complementary but are also partly correlated information.

### 4.3. Kelvin Waves

Kelvin waves from large and fast ships are occasionally observed in S2 images [7]. A sailing ship creates Kelvin waves bounded by cusp-lines separated by an angle of ±arcsin(1/3) = ±19.47° on each side of the ship and its wake. The Kelvin wavelength is related to the ship speed *V* as [17]

(16) $$\lambda =\frac{2\pi V^{2}}{g}$$

where *g* = 9.8 m/s^2^ is the gravitational acceleration at the surface of the Earth. The wavelength can be determined by a Fourier analysis of the image, and the ship speed follows from Equation (16).

**Figure 8:**
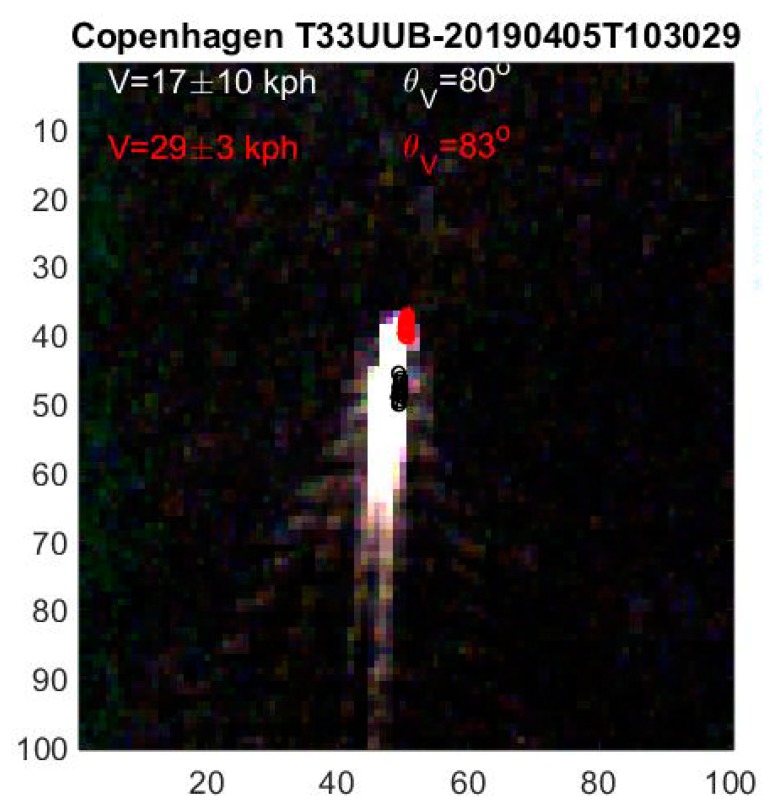
Ship with up to a dozen observable Kelvin waves in its wake.

In Figure 8, 10 Kelvin waves are observed within ca. 50 pixels, i.e., λ = 50 m, and we obtain the ship speed V = 8.8 m/s or 32 kph. The apparent ship speed from temporal delays is V = 29 ± 3 kph, when corrected for wakes. According to AIS, a ship at that position and time is sailing north with 30 kph in good agreement with both satellite results.

The Kelvin waves are stationary relative to the ship, i.e., travel with ship velocity as seen from the satellite. The time delay of 2 s between the first and last multispectral corresponds to ~20 m in this case or almost half a wavelength. The Kelvin wave in the last image is therefore in anti-phase with the first and tends to interfere destructively if the multispectral images are added or plotted on top of each other. The Fourier transform of the individual band images is therefore optimal. It also improves the transform if the ship is masked, which is straight forward to do as the ship central position, width, breadth and heading are known.

The time offsets also cause a Kelvin wave phase shift in the multispectral bands 

(17) $$\phi_{m}=\frac{2\pi Vt_{m}}{\lambda }=\frac{gt_{m}}{V}$$

Slow ships result in large phase shifts. The multispectral phases can also be found in the Fourier analysis. 

## 5. Discussion

The above results can be taken as proof of principle for our novel method utilizing the temporal offsets in the Sentinel-2 multispectral imager for determining velocities and altitudes of moving objects. For aircrafts in particular, the accuracy is excellent; a few kph compared to cruise velocities of the order of 1000 kph, and a few hundred meters uncertainty in altitude compared to the standard 10 km cruise altitude of commercial airliners. Contrails improve heading direction determination but can also debase the positions unless the threshold is set correctly between aircraft and contrail reflections. Jet streams when present were shown to affect velocities and altitudes significantly. Unfortunately, the jet stream velocity cannot be determined from the S2 images but requires separate atmospheric information or alternatively, the jet stream can be estimated by requiring the aircraft to fly with standard cruise speed or altitude of commercial airliners.

The method was also shown to apply to ships with similar uncertainty of a few kph, which is sufficient for fast ships but comparable to slow ships. However, wakes cause a serious systematic error as they corrupt the temporal towards spectral ordering. Utilizing measurements of ship length and adding them can correct for part of this error. Yet, a much better understanding of the correlations between wakes, speed and spectral reflections is required and needs further investigation.

Our method is limited in the sense that it only utilizes the central position and length of objects for each band. The image contains much more spectral information on the object extent and form that is not utilized. Yet, it works surprisingly well even for small and slow objects such as ships with a variety of complex wakes.

Moving objects over land was not considered in this work. The more complex background will require better algorithms for removing the background in each spectral image, which is outside the scope of this work.

## 6. Summary and Outlook

We have described the basic physics behind moving objects in satellite multispectral images with temporal offsets, parallax effects and influence of jet streams. The basic formulas were derived and as proof of principle, a number of representative examples were shown for aircrafts and ships. The analysis serves as a proof of principle and provides a working model.

From apparent velocities the resulting aircraft speed, heading, and altitudes were calculated accurately and compared to data from the navigation systems ADS-B with good agreement. Jet streams can influence aircraft speeds and altitudes and the jet stream velocity must be determined independently or fitted. 

Ship velocities are not affected by parallax but difficult to determine accurately for slow ship speeds or when long wakes are present. We described a simple but effective correction method, which improves the calculation of ship speeds considerably when compared to AIS. The detailed influence of thresholds, backgrounds, object lengths and contrails and wake reflections in the different multispectral bands should be studied in more detail in order to further improve the position determination. In addition, wake lengths may depend on e.g., ship type and size, surface winds and background. The temporal offsets are useful complementary information and the correlation to wake and ship lengths and widths provides additional information. Comparison to wake detection and velocity determination in Synthetic Aperture Radar radar images [18] should also be studied.

For better statistics, a large number of ships and aircrafts is required, where velocities and altitudes of aircrafts and ships are calculated and compared to AIS and ADS-B data with improved trajectory prediction [19]. This would also allow for improving the model by fine-tuning and optimizing parameters such as thresholds, better wake corrections and possibly introduce non-equal weights in the linear regression analysis of Equation (3). The large set of ships and aircrafts would also build an annotated database necessary for training machine learning algorithms [20,21,22,23]. Convolutional neural networks could be trained on this database so as to attempt to extract many more parameters and possibly refine the estimation of altitudes and velocities.

## Figures and Tables

**Table 1:** Spectral bands for the Sentinel-2B Multispectral Imager. The 10 high resolution bands *m =* 0, ..., 9 are ordered according to temporal offset [1].

S2 Spectral Band	Temporal Order (m)	Temporal Offset *t_m_* (s)	Central Wavelength (nm)	Bandwidth (nm)	Spatial Resolution (m)
**1**	-	2.314	442.2	21	60
**2**	0	0	492.1	66	10
**3**	2	0.527	559.0	36	10
**4**	3	1.005	664.9	31	10
**5**	4	1.269	703.8	16	20
**6**	6	1.525	739.1	15	20
**7**	7	1.790	779.7	20	20
**8**	1	0.263	832.9	106	10
**8a**	8	2.055	864.0	22	20
**9**	-	2.586	943.2	21	60
**10**	-	0.851	1376.9	30	60
**11**	5	1.468	1610.4	94	20
**12**	9	2.085	2185.7	185	20
